# Editorial: The microbiome in the development of gastrointestinal diseases

**DOI:** 10.3389/fcimb.2025.1561143

**Published:** 2025-03-13

**Authors:** Amedeo Amedei, Ralf Weiskirchen

**Affiliations:** ^1^ Department of Experimental and Clinical Medicine, University of Florence, Florence, Italy; ^2^ Network of Immunity in Infection, Malignancy and Autoimmunity (NIIMA), Universal Scientific Education and Research Network (USERN), Florence, Italy; ^3^ Institute of Molecular Pathobiochemistry, Experimental Gene Therapy and Clinical Chemistry (IFMPEGKC), RWTH University Hospital Aachen, Aachen, Germany

**Keywords:** microbiome, gastrointestinal disease, MAFLD, inflammatory bowel disease, colorectal cancer, dysbiosis, lipids, experimental models

**Figure 1:**
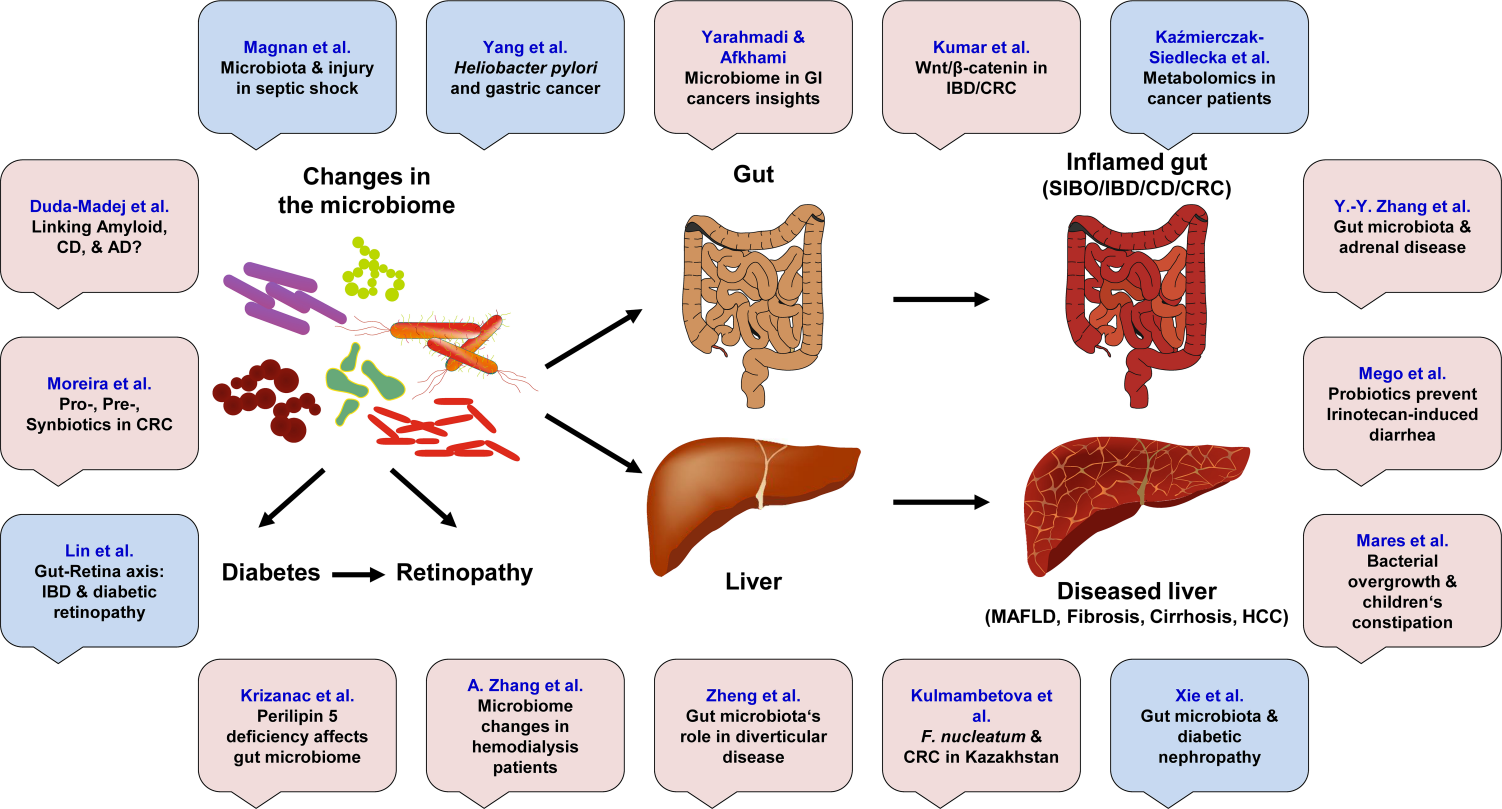
This Research Topic contains 11 original articles (marked in bright red) and 5 review article (marked in light blue). The articles highlight the relationships between changes in the gut microbiome and several gastrointestinal disorders, including colorectal cancer (CRC), inflammatory bowel disease (IBD), diverticular disease, small intestinal bacterial overgrowth (SIBO), metabolic dysfunction-associated fatty liver disease (MAFLD), and hepatocellular carcinoma (HCC). These changes can also be associated with diabetes, retinopathy, Alzheimer disease (AD) and many other disorders.

The human microbiome, a complex and dynamic ecosystem composed of trillions of microorganisms residing in various body sites, plays a critical role in maintaining health and homeostasis. Recent research has increasingly focused on the gut microbiome (GM) due to its significant influence on gastrointestinal (GI) health and its involvement in the development of various GI diseases. This editorial synthesizes findings from 16 manuscripts, including 11 original research articles, 3 reviews, 1 mini-review, and 1 systematic review, authored by a diverse group of 109 researchers from countries including China, Croatia, Finland, France, Germany, India, Iran, Kazakhstan, Poland, Portugal, Romania, Slovakia, and Sweden. Collectively, these studies highlight the intricate relationships between gut microbiome composition and several GI disorders, including colorectal cancer (CRC), inflammatory bowel disease (IBD), diverticular disease, small intestinal bacterial overgrowth (SIBO), and metabolic dysfunction-associated fatty liver disease (MAFLD) (**Figure 1**). The evidence presented reveals how dysbiosis (microbial communities’ imbalance) can contribute to inflammation, impaired immune responses, and altered metabolic functions that predispose individuals to these diseases. Furthermore, new insights into the gut-brain axis are revealing how GM can influence not only local intestinal health, but also systemic conditions affecting other organs. Interventions aimed at modulating the microbiome composition and/or function by prebiotics, probiotics, or dietary changes have shown promise in alleviating symptoms and improving treatment outcomes in patients with GI diseases. As our understanding of the GM role expands through this extensive body of work in multiple international contexts, it becomes increasingly clear that targeting the microbial balance may offer innovative strategies for the prevention and effective management of GI diseases.

The study by Magnan et al. investigates the relationship between GM, bacterial translocation, and acute GI injury in critically ill patients with septic shock. The study involved 60 adults over seven days and assessed changes in GM diversity and their correlation with clinical outcomes. Results show a significant decrease in bacterial diversity and richness from day 0 to day 7, with lower alpha diversity associated with higher SOFA scores. An increase in *Enterococcus* species was observed alongside a decrease in beneficial bacteria such as *Bifidobacterium*. In addition, increased levels of bacterial translocation were observed at both admission and day 7 compared to healthy controls (HC), suggesting that gut inflammation may promote bacterial translocation into the circulation. Mortality analysis revealed that non-survivors had lower GM diversity on admission. Certain genera such as *Mogibacteriaceae* were more abundant in non-survivors, while others such as *Escherichia* decreased over time. The increase in *Enterococcus* during hospitalization correlated with worse outcomes. The study concludes that dysbiosis and bacterial translocation significantly influence acute GI severity and mortality risk in septic shock patients, suggesting further exploration of therapeutic strategies targeting GM to improve patient outcomes.

Yang et al. present a bibliometric analysis of research related to *Helicobacter pylori* (HP) and gastric cancer (GC) from 2003 to 2022. Their study aims to assess scientific output, identify influential papers, summarize current knowledge, and explore emerging trends in the field. A total of 1,970 papers were retrieved, showing an increasing trend in publications over the years. China and Japan emerged as the leading contributors, with Vanderbilt University notable for its high output. Key authors include Richard M. Peek Jr. and Maria B. Piazuelo, both from Vanderbilt University. The journal “*Helicobacter*” published the most papers, while “*Gastroenterology*” had the highest number of citations. The analysis highlights relevant themes such as the HP role in gastric tumorigenesis, its pathogenesis in relation to GC and the mechanisms by which HP affects GC development. Emerging areas for future research include autophagy, GM interactions, implications for immunotherapy, exosome functions, epithelial-mesenchymal transition, and γ-glutamyl transpeptidase. The findings underscore that HP is a major risk factor for GC through mechanisms involving inflammation and immunity modulation. HP eradication may prevent early GC stages and improve treatment outcomes. In conclusion, this study provides valuable insights into the global landscape of HP/GC research suggesting potential directions for future investigations to address gaps in knowledge of their interplay.

The review by Yarahmadi and Afkhami highlights the significant link between GM and the development of GI cancers, which account for a third of new cancer cases worldwide. The authors discuss how perturbations in the GI microbiota may influence cancer progression, with some bacteria being cancer-promoting and others being protective. Recent studies suggest that alterations in GM composition are associated with several GI malignancies, including colorectal, gastric, liver and esophageal cancers. The review highlights the relevance of understanding these microbial communities and their interactions with the host immunity as potential avenues for cancer prevention and treatment strategies. The authors explore how GM can affect the efficacy of cancer therapies such as chemotherapy, immunotherapy, and radiotherapy. They report that dysbiosis can lead to inflammatory responses that exacerbate cancer progression and specific bacteria, such as *Fusobacterium nucleatum* (*F. nucleatum*), have been implicated in chemoresistance in CRC. Additionally, the authors discuss emerging research on non-bacterial components of the microbiome, including viruses and fungi, which also play a role in GI cancers. For example, certain viral infections have been associated with an increased cancer risk. In conclusion, this comprehensive analysis underscores the dual GM role in both facilitating and inhibiting GI carcinogenesis, while suggesting that modulation of these microbial communities may improve therapeutic outcomes for patients with GI cancers.

In their mini-review, Kumar et al. explore the relationship between GM, its secondary metabolites, and the regulation of the Wnt/β-catenin signaling pathway in the context of IBD and CRC. GI cancers represent a significant public health burden, with rising incidences associated with GM dysbiosis. The authors discuss how secondary metabolites produced by gut microbes, such as short-chain fatty acids (SCFAs) and bile acids, play a critical role in maintaining intestinal homeostasis and regulating inflammation-driven tumorigenesis. Dysbiosis can lead to altered levels of these metabolites, resulting in immune cells’ activation that contributes to chronic inflammation and increased cancer risk. The review emphasizes the relevance of the Wnt/β-catenin pathway in CRC progression; in detail its dual role in modulating inflammation and promoting cell proliferation. It highlights that microbial metabolites such as butyrate inhibit this pathway, suggesting potential therapeutic avenues for treating CRC through dietary or probiotic interventions. Additionally, the authors discuss how bile acids interact with nuclear receptors such as the farnesoid X receptor to influence both bile metabolism and the Wnt signaling pathway. This interplay provides opportunities for novel therapeutic strategies targeting these mechanisms to attenuate IBD-related inflammation and CRC development. In conclusion, understanding the interactions between gut-derived metabolites and Wnt signaling may provide new treatments approaches for GI cancers while minimizing the side effects associated with conventional therapies. The review calls for further research into these relationships in order to develop effective combinatorial therapies aimed at improving treatment outcomes for patients with IBD and CRC.

The research article by Kaźmierczak-Siedlecka et al. investigates the gut metabolome in patients with gastric cancer (GC) (n=4) and CRC (n=8) prior to initiation of anticancer treatments. The study aims to explore potential differences in metabolite profiles that could serve as biomarkers for these cancers. Stool samples were collected from 12 patients, and untargeted metabolomics was performed using mass spectrometry to analyze a wide range of metabolites. The results revealed distinct metabolic profiles, with higher levels of certain metabolites found predominantly in CRC patients compared to those with GC. Notably, metabolites such as deoxyguanosine, uridine, L-phenylalanine, and 3-indoleacetic acid were significantly elevated in CRC patients. The analysis revealed a more homogeneous metabolic profile among GC patients compared to the diverse profiles observed in CRC patients. This suggests that tumor localization may influence the GM activity and so its metabolites’ production. The authors acknowledge the limitations due to the small sample size but emphasize that these preliminary findings pave the way for further research into untargeted metabolomics as a non-invasive tool for early detection and monitoring of GI cancers. Future studies are planned to assess the impact of anti-cancer treatments on these metabolic profiles. In conclusion, this study highlights the potential role of gut-derived metabolites as biomarkers for discriminating between GC and CRC, while highlighting the need for larger studies to validate these findings.

The review by Duda-Madej et al. explores the potential links between Crohn’s disease (CD), a chronic inflammatory bowel condition, and Alzheimer’s disease (AD), a common neurodegenerative disorder. Both diseases are characterized by complex pathomechanisms involving genetic, environmental, immunological, and microbiological factors. Recent evidence suggests that chronic inflammation in conditions such as CD may increase the risk of AD developing. The authors highlight the gut-brain axis as a critical pathway linking these two diseases, where GM influences neuroinflammatory processes and amyloid aggregation associated with AD. Specifically, they discuss how GM dysbiosis can lead to increased permeability of the intestinal barrier, allowing pro-inflammatory substances to enter the systemic circulation and potentially reach the brain. The review emphasizes the role of amyloid proteins produced by both human cells and gut bacteria, especially bacterial amyloid peptides (curli fimbriae) from certain bacteria that mimic human amyloids. These bacterial amyloids may contribute to neuroinflammation and amyloid-beta aggregation in AD. Furthermore, the authors note that alterations in the GM composition may affect immune responses and metabolic processes associated with both CD and AD. They call for further research into microbial metabolites as potential therapeutic targets for the treatment of both diseases. In conclusion, this review suggests a significant relationship between CD and AD through shared inflammatory pathways and microbial influences. Understanding these links may lead to novel strategies for the prevention and treatment of neurodegenerative diseases rooted in GI health.

In a systematic review by Moreira et al. the therapeutic potential of GM modulation by prebiotics, probiotics, and synbiotics in patients with CRC is discussed. The review follows PRISMA guidelines and includes 24 randomized controlled trials (RCTs) assessing the effects of these supplements on CRC treatment outcomes, focusing on surgical recovery, chemotherapy, and radiotherapy side effects. The authors found that supplementation significantly improved surgical outcomes by decreasing postoperative complications, such as infections and GI symptoms like diarrhea. The results showed that patients who received probiotics or synbiotics had a faster return to normal gut function and shorter hospital stays than control groups. In detail, specific strains such as *Lactobacillus rhamnosus* and *Bifidobacterium lactis* were often associated with positive outcomes. However, the evidence regarding the optimal formulations, such as strain combinations, dosages, and administration duration, remains limited due to high heterogeneity between trials. In addition, the review highlights that while some trials reported benefits of probiotic supplementation during chemotherapy or radiotherapy, others showed no significant improvements. The authors emphasize the need for more RCTs with larger sample sizes and standardized protocols to further clarify the effectiveness of these interventions. In conclusion, this systematic review suggests that pre-, pro-, and synbiotic supplementation may offer beneficial effects for CRC patients undergoing treatment by improving recovery and alleviating treatment-related side effects. Future research should focus on optimizing these interventions to improve clinical outcomes in CRC management.

The research article by Lin et al. investigates the potential association between IBD and diabetic retinopathy (DR) using Mendelian randomization (MR) and mediation analysis. The study uses genome-wide association study (GWAS) data to explore causal relationships, focusing on IBD subtypes, ulcerative colitis (UC) and CD, and their association with DR. The results indicate a significant negative correlation between UC and DR risk, suggesting that increased inflammation in IBD may affect retinal health. Conversely, the authors suggest that DR may reduce the CD incidence. Mediation analysis identified circulating inflammatory proteins, in particular fibroblast growth factor 21 (FGF21), phosphatidylcholine, and triglycerides, as mediators in these relationships. Elevated FGF21 levels are associated with both microvascular complications in diabetes and intestinal inflammation, highlighting its potential role as a biomarker for DR. The authors emphasize the relevance of understanding the gut-retina axis, noting that dysbiosis in DR may affect systemic inflammation and lipid metabolism, influencing both conditions. They acknowledge limitations such as the focus on participants of European ancestry in the GWAS data, which may affect generalizability. In conclusion, this research provides insights into common pathways between IBD and DR, suggesting that therapeutic strategies targeting these pathways may improve outcomes for patients with both conditions. Further studies are warranted to explore these relationships more comprehensively in diverse populations.

Mares et al. investigate the relationship between SIBO and constipation in pediatric patients. SIBO is characterized by an abnormal increase in bacteria in the small intestine, leading to symptoms ranging from mild GI discomfort to more serious problems such as malabsorption. The authors conducted a thorough literature search and included 79 studies that investigated the prevalence, diagnosis, and treatment of SIBO in children. They highlighted the challenges of diagnosing SIBO due to variations in methodology and lack of standardized criteria, with particular emphasis on breath tests using glucose or lactulose as substrates. The findings suggest that SIBO is common in children with functional GI disorders, although rates vary widely depending on study design. The review notes a strong association between methane production during breath testing and constipation, although results are inconsistent between studies. Treatment strategies for SIBO typically include antibiotics, dietary changes, and probiotics, but research into pediatric applications remains limited. The authors emphasize the need for well-designed studies with larger sample sizes to establish clearer diagnostic criteria and effective treatment protocols tailored for children. In conclusion, although there is evidence linking SIBO to constipation in children, further research is needed to clarify these associations and improve clinical management strategies. The article calls for future research to focus on standardized diagnostic methods and to explore dietary interventions or probiotic therapies as potential treatments for pediatric SIBO.

In their original article, Xie et al. investigated the potential causal relationship between GM and diabetic neuropathy (DN) using MR. The study aimed to clarify how GM changes may influence the DN development and vice versa. Data from GWAS were used, focusing on non-Finnish Europeans for IBD and the FinnGen project for DN. The authors used various MR methods, including inverse-weighted variance analysis, to determine causal relationships while assessing pleiotropy and heterogeneity. The results showed significant associations between specific GM taxa and DN. Elevated levels of the *Christensenellaceae R-7*, *Ruminococcaceae UCG013*, and *Eggerthella* groups were associated with an increased DN risk, whereas *Peptococcaceae and Eubacterium coprostanoligenes* showed protective effects. Reverse MR analysis revealed that elevated levels of *Anaerofilum*, *Dorea*, *Lachnospiraceae UCG-010*, *Ruminococcus 2*, and order NB1n may also contribute to an increased risk of DN. The study highlights the importance of understanding the gut-retina axis in relation to metabolic diseases such as diabetes. It suggests that GM-derived metabolites could serve as non-invasive diagnostic or therapeutic targets for early DN detection. In conclusion, this research provides valuable insights into the complex interactions between GM and DN and supports the hypothesis that these factors are causally related. Further investigations are needed to elucidate the underlying mechanisms and to explore potential clinical applications in the treatment of DN by GM modulation.

The article by Zhang et al. examines the association between GM and three adrenal disorders: adrenocortical insufficiency (AI), Cushing’s syndrome, and hyperaldosteronism (HA). Using data from GWAS, the authors used a bi-directional MR approach to investigate these associations. The study identified several bacterial taxa associated with AI, such as *Deltaproteobacteria* and *Desulfovibrionaceae*, which were found to have protective effects against AI. On the other hand, certain families including *Porphyromonadaceae* were associated with an increased AI risk. *Acidaminococcaceae* was identified as a protective factor for Cushing’s syndrome, while *Methanobacteria* and *Lactobacillaceae* played a protective role in hyperaldosteronism. Conversely, genera such as *Parasutterella* and *Peptococcus* were associated with an elevated risk for HA. Sensitivity analyses confirmed the reliability of these results, showing no significant horizontal pleiotropy or heterogeneity between the instrumental variables used in the MR analysis. The inverse MR analysis showed no significant causal relationships from adrenal disease to GM. The authors suggest a potential causal relationship between specific gut microbial taxa and adrenal disease, which may provide new diagnostic opportunities and focus on GM modulation. However, they acknowledge limitations related to sample diversity and the exclusion of many single nucleotide polymorphisms during their analysis, a call for further research to validate these findings in diverse populations.

The study by Mego et al. evaluates the probiotics’ effectiveness in reducing irinotecan-induced diarrhea in CRC patients with colostomies. The analysis combines data from two clinical trials involving 279 patients, who were randomized to receive either probiotics or a placebo during their chemotherapy regimen. The results show that while the overall incidence rates of grade 3/4 diarrhea did not differ significantly between groups, subgroup analyses showed that patients with colostomies who received probiotics had a significantly lower incidence of any diarrhea (25.7% *vs*. 51.2%, *p*=0.028) and no cases of severe diarrhea compared to those who received placebo. Probiotic use was also associated with reduced use of anti-diarrheal medication, although this finding was not statistically significant. The study highlights the potential probiotics’ benefits specifically for CRC patients with colostomies undergoing treatment with the topoisomerase inhibitor irinotecan, and suggests that maintaining a healthy GM could help to reduce GI toxicity associated with chemotherapy. Despite these promising findings, the authors acknowledge limitations such as the variability of probiotics’ formulations and the lack of preclinical testing before human trials. They call for further research into different probiotic strategies and the mechanisms underlying the observed effects to optimize treatment outcomes for CRC patients experiencing chemotherapy-related side effects. Overall, the study points to a critical area for future investigation regarding GM modulation in cancer treatment.

In their original article, Krizanac et al. investigated the role of perilipin 5 (PLIN5) in regulating GM during the development of metabolic dysfunction-associated fatty liver disease (MAFLD) and its progression to hepatocellular carcinoma (HCC). Using mouse models, the study investigated how PLIN5 deficiency affects the GM composition when mice are fed a Western diet. The authors observed significant changes in microbial diversity, with an increased abundance of beneficial taxa such as *Lactobacillus* in *Plin5*-deficient mice compared to wild-type controls. Additionally, the study found that a Western diet exacerbated these microbial changes and specific bacterial taxa associated with metabolic pathways relevant to liver health were identified. *Deltaproteobacteria* and *Desulfovibrionaceae* were associated with protective effects against liver disease, while certain other taxa were associated with an increased disease risk. The results suggest that *Plin5* plays a critical role in shaping the GM composition, thereby influencing metabolic processes associated with liver disease. By highlighting the interactions between dietary factors, genetic predisposition, and GM, this research opens avenues for potential therapeutic strategies targeting *Plin5* to modulate gut flora and mitigate the progression of liver disease. In conclusion, the study highlights the relevance of understanding how *Plin5* deficiency affects GM dynamics within MAFLD and subsequent progression to HCC, and emphasizes future research directions focusing on human studies to further explore these relationships for therapeutic applications.

The original paper by Zhang et al. investigates the GM profiles in maintenance hemodialysis (MHD) patients who suffer from constipation. They aim to understand how changes in gut flora may contribute to GI symptoms commonly observed in these patients. Fecal samples were collected from 45 participants, including 15 with MHD-related constipation, 15 without constipation, and 15 healthy controls. The authors analyzed differences in GM composition between the groups. The results showed that the MHD constipation group had reduced microbial diversity compared to non-constipated MHD patients and healthy controls. At the genus level, *Enterococcus* and *Escherichia-Shigella* were dominant in constipated patients, while beneficial taxa such as *Bifidobacterium* and *Faecalibacterium* were less abundant. The analysis suggested that certain potentially pathogenic bacteria may exacerbate inflammation and contribute to constipation. Additionally, pathways involved in pyruvate metabolism and flavonoid biosynthesis were enriched in constipated patients, suggesting a metabolic dysregulation associated with their condition. The study highlights a potential link between GM composition and inflammatory responses that may influence gut function. In conclusion, this research provides insights into how GM changes iome may influence constipation in MHD patients. It highlights the need for further investigation into specific bacterial taxa and their role in GI health to develop targeted therapeutic strategies, such as fecal microbiota transplantation (FMT), for the management of constipation associated with hemodialysis.

Zheng et al. investigated the causal relationship between GM and intestinal diverticular disease using a bidirectional two-sample MR approach. The study used genetic instrumental variables from a genome-wide association study involving 5,959 participants to assess the GM effect on diverticular disease, including 5,193 cases and over 457,000 controls sourced from the IEU Open GWAS project. The analysis revealed significant associations between specific microbial taxa and the risk of developing intestinal diverticular disease. In detail, increased levels of *Caryophanales*, *Paenibacillaceae*, *Herbinix*, *Turicibacte*r, and *Staphylococcus fleurettii* were associated with a higher risk of the disease. Conversely, *Chromatiales* and *Arcobacter* showed protective effects. In addition, reverse MR analysis did not reveal any significant causal relationships from diverticular disease back to GM. The findings underscore that variations in GM composition may influence the onset and progression of diverticular disease. This research highlights the importance of understanding how GM changes may contribute to GI disorders such as diverticulosis. It suggests potential avenues for personalized treatment strategies targeting specific microbial populations to prevent or effectively treat diverticular disease. In conclusion, this study provides insights into the relationship between GM and diverticular disease, while highlighting the need for further research in diverse populations to validate these findings and more fully explore the underlying biological mechanisms.

The original research article by Kulmambetova et al. investigates the prevalence of *F. nucleatum* and its association with CRC in patients in Kazakhstan. The study included 83 patients with histologically confirmed CRC, from whom 249 biopsy specimens were collected, including carcinoma tissue (CT), adjacent normal tissue (AT), and distant normal tissue (NT). Using quantitative real-time polymerase chain reaction, the authors detected *F. nucleatum* along with other CRC-associated bacteria such as *Bacteroides fragilis*, *Escherichia coli*, and *Streptococcus gallolyticus*. The results showed a significantly higher *F. nucleatum* prevalence in CT compared to AT and NT, with detection rates of 43.4%, 27.7% and 24.1%, respectively (*p*=0.02). The frequency of *F. nucleatum* was significantly higher in tumors located distally in the colon and was associated with larger tumor size and higher consumption of processed meat. Additionally, although no significant correlations were found between *F. nucleatum* infection and various clinical characteristics such as age or sex, the study highlights its potential as a marker for CRC diagnosis due to its association with tumor progression. In conclusion, this study underscores the role of *F. nucleatum* in the CRC pathogenesis in Kazakhstani patients and suggests that it may serve as a valuable diagnostic biomarker for CRC management, warranting further investigation into its mechanisms and implications for cancer development.

The collection of reviews and original research articles highlights the significant role of the GM in various health conditions, particularly in relation to GI and metabolic diseases. Studies investigating the associations between GM and conditions such as CRC, diabetic neuropathy, diverticular disease, and complications arising from maintenance hemodialysis provide compelling evidence that microbial composition can influence disease development, progression, and treatment outcomes. For example, the systematic review of pre-, pro- and synbiotic supplementation suggests that these interventions may improve surgical outcomes and reduce chemotherapy-related side effects in CRC patients. Similarly, research into *F. nucleatum* shows its prevalence in CRC tissues and its potential as a diagnostic marker for this malignancy. Furthermore, studies using MR demonstrate causal relationships between GM changes and various diseases, reinforcing the concept that specific microbial taxa may either contribute to or protect against diseases such as DN and diverticular disease. Overall, these findings underscore the relevance of understanding the dynamics of the gut microbiome and its interactions with host physiology. They suggest that targeting GM profiles through dietary modification or probiotic therapies may offer promising avenues for prevention and management strategies in various health contexts. This growing body of evidence highlights the need for continued research into the complex interplay between GM, diet, inflammation, and disease in order to develop effective therapeutic approaches aimed at restoring microbial balance for improved health outcomes. In particular, potential confounding factors such as diet, lifestyle, genetic predisposition and environmental exposures play a critical role in shaping the GM composition and function. A more nuanced understanding of how these elements interact with microbial communities can help elucidate their collective impact on health outcomes and disease susceptibility.

